# Supplementary material for: Deletion of wfs1 Impairs Oligodendrocyte Precursor Cells Dorsal Distribution and Myelination Through the wfs1-hmgcs1 Axis in Zebrafish
Source: Biology (Basel). 2026 Aug 16;15(16):1402. doi: 10.3390/biology15161402 (PMC13510151; doi:10.3390/biology15161402)
Supplement: Supplementary file 1 [file biology-15-01402-s001.zip › File S1-Transcriptome sequencing data and differentially expressed genes (DEGs) in wfs1-- zebrafish at 5 dpf/2.KEGG/wfs1KOvsWT/ALL/src/dre00400.html]

dre00400


- dre:561410

- Up regulated genes

561410\_1(1.20432290851387)

- dre:561410

- Up regulated genes

561410\_1(1.20432290851387)

- dre:406688

- Down regulated genes

406688(-1.12341806422973)

- dre:406688

- Down regulated genes

406688(-1.12341806422973)

Close
